# Developing T Cell Epitope-Based Vaccines Against Infection: Challenging but Worthwhile

**DOI:** 10.3390/vaccines13020135

**Published:** 2025-01-28

**Authors:** Xian Tang, Wei Zhang, Zheng Zhang

**Affiliations:** 1 The Second Affiliated Hospital, School of Medicine, Southern University of Science and Technology, Institute for Hepatology, National Clinical Research Center for Infectious Disease, Shenzhen Third People’s Hospital, Shenzhen 518112, China; tangxian@mail.sustech.edu.cn (X.T.); 12031106@mail.sustech.edu.cn (W.Z.); 2 Guangdong Key Laboratory for Anti-Infection Drug Quality Evaluation, Shenzhen 518112, China

**Keywords:** T cell epitope, vaccine development, infection

## Abstract

T cell epitope-based vaccines are designed to elicit long-lived pathogen-specific memory T cells that can quickly activate protective effector functions in response to subsequent infections. These vaccines have the potential to provide sustained protection against mutated variants, such as severe acute respiratory syndrome coronavirus 2 (SARS-CoV-2), which are increasingly capable of evading neutralizing antibodies. Recent advancements in epitope discovery, T cell receptor analysis, and bioinformatics have enabled the precise selection of epitopes and the sophisticated design of epitope-based vaccines. This review outlines the development process for T cell epitope-based vaccines. We summarize the current progress in T cell epitope discovery technologies, highlighting the advantages and disadvantages of each method. We also examine advancements in the design and optimization of epitope-based vaccines, particularly through bioinformatics tools. Additionally, we discuss the challenges of validating the accurate processing and presentation of individual epitopes and establishing suitable rodent models to evaluate vaccine immunogenicity and protective efficacy.

## 1. Introduction

Vaccines are considered one of the safest and most effective ways to prevent infectious diseases and save lives. Most vaccines induce neutralizing antibodies and have played a key role in combating diseases such as diphtheria, measles, and polio. However, challenges remain with certain pathogens [1,2]. Human immunodeficiency virus (HIV) and hepatitis C virus (HCV) present unique obstacles in developing protective vaccines, primarily due to their significant genetic diversity and ability to evade host immune responses [3,4]. There is a growing consensus that a strong T cell response is essential for effective vaccines against HIV and HCV [5,6,7]. While current vaccines against the hepatitis B virus (HBV) effectively induce neutralizing antibodies against HBV surface antigens (HBsAg) and protect healthy individuals from infection, progress with therapeutic HBV vaccines has been limited. These therapeutic vaccines aim to enhance T cell responses to target and eliminate infected liver cells in patients with chronic hepatitis B (CHB) [8,9]. Additionally, controlling intracellular bacteria through antibody-mediated immunity is challenging. Bacillus Calmette Guérin (BCG), the only available vaccine against tuberculosis (TB), stimulates protective antibody responses but provides limited and variable protection against *M. tuberculosis* [10,11,12]. More efforts are being directed toward developing TB vaccination strategies that focus on long-term memory T cell responses [13,14]. Available vaccines against SARS-CoV-2 have been broadly effective at reducing morbidity and mortality, with neutralizing antibodies believed to be key immune effectors in providing protection [15,16]. However, the rapid decline in neutralizing antibodies after infection or vaccination, combined with the continuous emergence of highly divergent viral variants, significantly impacts the durability of antibody-mediated protection [17,18,19,20]. This situation underscores the importance of T cell immunity in developing vaccines that can withstand viral mutations and provide long-lasting protection [21].

Unlike antibodies, which interact with extracellular forms of pathogens and their secreted products, T cells recognize foreign antigens derived from pathogens that replicate inside cells or are internalized through endocytosis. As a result, T cells are effective at clearing intracellular pathogens that are inaccessible to antibodies. While antibodies recognize native proteins and can be affected by mutations that alter the antigen’s structure, T cells target digested peptides representing conserved regions of pathogens, making them resistant to antibody escape variants [22,23]. Antibodies bind directly to intact antigen proteins, whereas T cells recognize epitopes presented by major histocompatibility complex (MHC) molecules on the surface of antigen-presenting cells (APCs). The polygeny and polymorphism of MHC molecules enhance T cells’ ability to respond to a wide variety of rapidly evolving pathogens by presenting a broad range of epitopes [24]. Therefore, developing T cell-based vaccines that effectively induce long-term memory T cells may be a reasonable and effective strategy for providing persistent protection against constantly mutating viruses and intracellular bacteria [25]. Since T cells specifically recognize short amino acid sequences rather than whole antigens, designing immunogens based on epitope sequences allows for excluding non-immunogenic protein fragments and potentially toxic or immunosuppressive viral components [26].

## 2. T Cell Epitope Discovery

**Figure 1:**
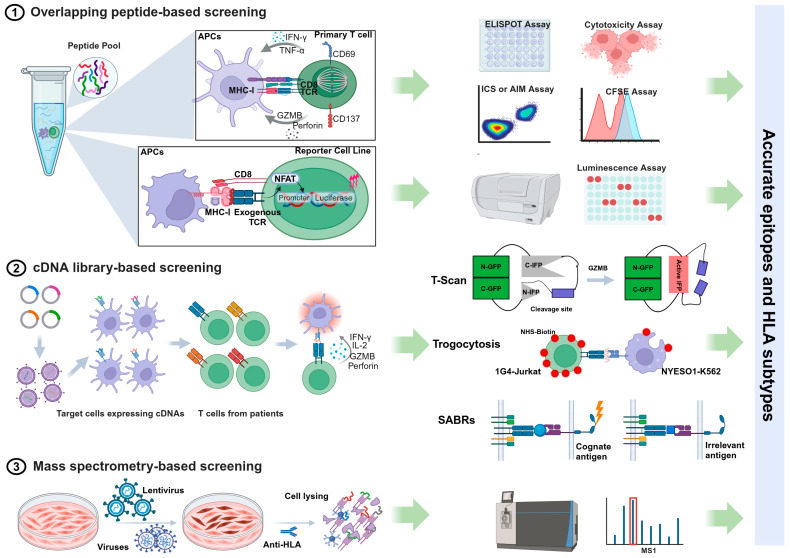
Summary of workflows and assay methods of different T cell antigen discovery approaches.

Screening and identifying pathogen epitopes recognized by T cells in response to infections is essential for developing epitope-based vaccines. However, despite significant research and technological advancements (Figure 1), epitope discovery remains a challenging and labor-intensive task due to the nature of T cell recognition (Table 1). The vast diversity of the T cell receptor (TCR) repertoire means that specific T cell clones for a given antigen are often present in very low numbers. Additionally, the affinity of TCR-pMHC interactions is much lower compared to the interactions between antibodies and antigens, making it difficult to detect these scarce populations of interest [27,28,29]. Moreover, antigen processing and presentation generate a wide variety of peptides that must be synthesized or expressed in antigen-presenting cells (APCs) for screening T cell epitopes [30,31]. Furthermore, the high degree of TCR cross-reactivity complicates matters, as a single TCR can bind to as many as 1 million different epitopes presented by polymorphic HLA molecules [32,33,34].

### 2.1. Overlapping Peptide-Based Screening

An effective and widely accepted approach for identifying epitopes is screening antigen-specific T cells using pools of synthetic overlapping peptides, each 15 amino acids long. The enzyme-linked immunospot (ELISpot) assay and intracellular cytokine staining (ICS) are commonly used methods to detect cytokine production by T cells in response to peptide stimulation [35,36]. Other functional measures of antigen-specific responses include assessing the proliferative capacity (CSFE assay), degranulation (CD107a), cytotoxic ability, and the upregulation of activation-induced markers (AIM assay) [37]. Alternatively, T cell receptors (TCRs) from a population of T cells can be analyzed using single-cell sequencing technologies. These TCRs are cloned and expressed on the surface of a TCR-negative T cell line equipped with a nuclear factor of activated T cells (NFAT)-luciferase reporter system [38]. When the TCR recognizes an antigen, it triggers luminescence, facilitating the discovery of epitopes through measurements of TCR-mediated activation. Emerging technologies that implement single-cell functional studies enhance the identification and isolation of antigen-specific T cells. For example, the Lightning™ optofluidic platform can perform T cell functional assays while simultaneously recovering antigen-responsive cells for downstream TCR analysis [39,40].

While this systematic screening approach allows for the precise identification of immunogenic peptides without requiring knowledge of the donor’s HLA profile or peptide–MHC binding characteristics, it is a time-consuming and costly process. This method involves synthesizing a large number of peptides. Specifically, for pathogens with large genomes, such as tuberculosis (TB), it is impractical to use overlapping peptides for the entire genome. Additionally, the overlapping peptide system does not account for non-canonical or cryptic epitopes that may arise from unexpected events during transcription and translation. These events can include mutations in non-coding intronic sequences [41], the improper splicing of introns [42,43], and ribosome frameshifting [44]. Moreover, epitopes generated by post-translational modifications (PTMs)—a natural covalent process necessary for properly folded and functional proteins—cannot be detected using synthetic peptide pools, particularly in contexts such as infection, tumors, or autoimmunity [45,46,47].

### 2.2. cDNA Library-Based Screening

Using endogenous protein processing and presentation through complementary DNA (cDNA) libraries that encode full-length open reading frames (ORFs), protein fragments, or short peptides significantly increases the variety of identified epitopes compared to synthesized peptides. Unlike MHC class I antigens, which are typically 8–11 amino acids in length, MHC class II epitopes are more diverse in size, usually ranging from 13 to 25 residues [48]. This variability makes predicting them for chemical synthesis challenging. cDNA libraries help address these length constraints and provide a valuable method for identifying CD4^+^ T cell epitopes [30,49]. Additionally, endogenous antigen expression enables the identification of proteasome-generated spliced peptides. These peptides are not derived from the genome; instead, they are formed through the cleavage and ligation of segments from the same protein (cis-splicing) or from two different proteins (trans-splicing) [50,51]. This comprehensive approach facilitates genome-wide screening for T cell antigens by combining single-cell functional readouts with deep sequencing technology.

Innovative cell-based assay methods have emerged in recent years, enabling the marking and isolation of target cells that express T cell antigens for deep sequencing. T-scan is a genome-wide platform for epitope mapping of CD8^+^ T cells. It works by utilizing specific protein cleavage in target cells carrying the epitope, triggered by granzyme B (GzB) when recognized by T cells with the corresponding TCR [52]. A few years later, a similar approach called T-scan II was developed by the same research group to identify CD4^+^ T cell antigens [53]. More recently, they published a new method named TCR Mapping of Antigenic Peptides (TCR-MAP), which leverages the interaction between CD40 and CD40 ligand (CD40L) expressed on engineered T cells and their target antigen-presenting cells (APCs) that express processed peptides. This interaction triggers Staphylococcus aureus transpeptidase (SrtA)-mediated tagging of the APCs [54]. Another innovative strategy employs the principle of trogocytosis, a biological phenomenon where plasma membrane fragments are transferred from the presenting cell to the lymphocyte when the two cells are conjugated and form an immunological synapse [55,56]. Additionally, chimeric receptors known as Signaling and Antigen-Presenting Bifunctional Receptors (SABRs) are designed to encode MHC-I or MHC-II molecules (SABR-IIs). These receptors present an extracellular pMHC complex attached to an intracellular TCR-like signal transducer, enabling the detection of TCR-pMHC interactions for antigen discovery [57,58].

However, it is important to note that cell lines and other in vitro systems may not fully replicate the natural antigen processing machinery and MHC presentation pathways found in APCs at different anatomical sites during infection and inflammation. As a result, these systems may either miss or over-represent epitopes that are generated in vivo [59,60,61,62].

### 2.3. Mass Spectrometry-Based Screening

Since a T cell epitope must bind to an MHC molecule, characterizing MHC-bound peptides from pathogen-infected cells using mass spectrometry (MS)-based techniques is a strategy for discovering naturally processed and presented candidate epitopes [63,64]. Immunopeptidomic analysis of cell lines reduces the need for large volumes of primary samples in immunogenicity assays [62]. This approach also enables the identification of unconventional peptides in the context of infection or tumors, including extended peptides that go beyond the HLA binding groove [65,66,67], as well as out-of-frame and post-translational modification (PTM) epitopes [68,69,70]. Although MS-based analysis has rapidly advanced and become a powerful technique for T-cell epitope discovery in the past decade, it often fails to identify less abundant antigens. Furthermore, peptides naturally presented on MHC molecules are not necessarily immunogenic; the immunogenicity of an antigenic peptide depends not only on how well it binds to MHC molecules but also on its conformation when bound [71,72]. Therefore, MS approaches may yield false positives and false negatives, necessitating further validation through immunogenicity screening [30].

### 2.4. Bioinformatics Tools for Epitope Prediction

To enhance efficiency and reduce time and costs in vaccine development, bioinformatics approaches have been applied, primarily focusing on selecting appropriate antigens and designing vaccines [73]. Immunoinformatics is a branch of bioinformatics that uses mathematical and computational techniques to analyze and interpret immunological data, allowing for predictions of immune responses to specific molecules [74]. For the design and development of T cell epitope-based vaccines, numerous immunoinformatic databases are available to search for epitopes that can bind to MHC molecules. These databases include IEDB, NetCTL, MHCPred, NetMHC, nHLAPred, CTL-Pred, SVMHC, RANKPEP, BIMAS, MAPPP, ProPred, SYFPEITHI, PREDEP, and MHCPEP [75,76]. The methodology of analyzing a pathogen’s genome to discover potential antigens is referred to as “reverse vaccinology” [74].

Another important immunoinformatic strategy is “structural vaccinology”, which provides a three-dimensional perspective on vaccine development. This approach focuses on the conformational properties of macromolecules and uses computational techniques from the field of structural biology to predict potentially strong binding interactions [77]. High-performance bioinformatics tools, such as molecular dynamics (MD) simulations, can be employed to select appropriate peptides capable of binding and forming stable complexes with MHCs [78]. Major software packages for MD simulations include AMBER, CHARMM, GROMACS, LAMMPS, and NAMD [79,80,81,82,83]. Molecular docking is another quick and powerful technique for investigating intermolecular interactions. It has been utilized to select MHC binders with optimal shape complementarity and minimal binding energy. Widely used tools for molecular docking include UCSF Chimera v1.11.2, OpenBabel, and AutoDock Vina v1.2.0. [84,85,86].

## 3. Epitope-Based Vaccine Development

Traditionally, six categories of vaccines show promise in stimulating strong T cell responses: live attenuated vaccines, replication-competent and replication-defective recombinant live-vectored vaccines, DNA vaccines, mRNA vaccines, and heterologous prime-boost vaccines [87]. With advancements in epitope discovery technologies and our growing understanding of T cell responses, vaccine design and delivery have become more sophisticated. The precise selection of peptide epitopes that represent the minimal immunogenic regions as vaccine components allows for accurate and effective targeting of immune responses [72].

### 3.1. Synthetic Peptide Vaccines

Peptide-based vaccines that utilize synthetic peptides to activate epitope-specific T cells offer several advantages [72,88]. They are generally safer than other types of vaccines because they contain minimal components of T cell antigens and exclude sequences that could cause side effects. There is no risk of virulence reversion or the integration of DNA into the host cell genome, which are concerns with live attenuated and DNA vaccines, respectively. Additionally, peptide vaccines are easy to produce, quick to manufacture, and cost-effective, allowing for large-scale production. Lyophilized peptides can be stored, transported, and distributed at room temperature, eliminating the need for cold chain facilities [72,89]. For example, a peptide vaccine containing a single CD4^+^ T cell epitope from the *Salmonella*-secreted effector protein I (SseI) provided significant protection to susceptible mice against lethal *Salmonella* infection [90]. More recently, three doses of a peptide vaccine containing a single CD8^+^ T cell epitope conferred protection against lethal SARS-CoV-2 infection in the K18-hACE2 transgenic mouse model, even in the absence of neutralizing antibodies [91].

A key limitation of vaccines based on T cell epitopes is the tendency of viral mutations in these epitopes to evade the immune response, particularly with highly mutated viruses such as HIV and HCV [7,92,93,94]. Furthermore, the use of epitope-based vaccines is often restricted to patients with specific HLA haplotypes. To counter potential immune evasion and improve HLA coverage, a simple physical mixture of peptides is frequently used [89]. The HCV peptide vaccine IC41, composed of five synthetic T cell epitope peptides, induced HCV-specific T cell responses in humans [95]; however, it did not prevent HCV-RNA relapse in patients undergoing ongoing interferon treatment [96]. More recently, CoVac-1, a peptide vaccine consisting of six HLA-DR-restricted SARS-CoV-2 peptides, elicited multifunctional CD4^+^ and CD8^+^ T cells in all study participants [97].

While T cell epitopes are central to initiating an immune response by providing antigen stimulation (Signal 1), they are not sufficient on their own. Co-stimulation (Signal 2) and cytokine signals (Signal 3) are also crucial for enhancing T cell activation and differentiation. Recent findings indicate that nutrients (Signal 4), such as glucose, amino acids, and lipids, play important roles in regulating T cell responses and interact with Signals 1–3 to promote T cell immunity [98,99]. Therefore, using strong immunostimulant adjuvants and effective delivery systems is essential for vaccine peptides, which often have weak immunogenicity and are susceptible to enzymatic degradation, to elicit a potent immune response [72]. Immunostimulants, including Toll-like receptor (TLR) agonists, pathogen-associated molecular patterns (PAMPs), damage-associated molecular patterns (DAMPs), cytokines, and various nutrients and metabolites, activate the innate immune system and promote the activation and maturation of dendritic cells (DCs), leading to a robust T cell response [100,101,102]. Delivery systems such as lipid nanoparticles (LNPs), poly(lactic-co-glycolic acid) (PLGA), and caged protein nanoparticles help protect protease-sensitive peptides from degradation and facilitate their uptake by antigen-presenting cells (APCs) [103,104].

### 3.2. Multi-Epitope Vaccines

In nature, pathogens present various antigens with multiple epitopes. Therefore, a multi-epitope vaccine composed of a series of epitopes is an ideal approach for inducing T cell responses across these epitopes. Although combinations of specific peptides have successfully induced T cell responses in humans and protected mice from viral challenges, vaccination with a CTL epitope derived from the human adenovirus type 5 E1A region (Ad5E1A234-243) actually enhanced tumor growth rather than inhibiting it, due to CD8+ T cell tolerance [105]. Subsequent studies have shown that this tolerance occurs when short peptides are directly loaded onto HLA molecules on non-dendritic cells (non-DCs), which lack the co-stimulatory molecules necessary for generating effective effector T cells [106,107]. In contrast, longer peptides are preferentially presented by professional antigen-presenting cells (APCs), which helps prevent CD8^+^ T cell tolerance and increases the overall magnitude of the T cell response [108,109]. Synthetic long peptide (SLP) vaccines have emerged as a straightforward solution, as they require processing and are exclusively presented by professional APCs [110]. A significant improvement in the immunogenicity of peptide vaccines against HIV and HCV has been achieved by physically linking CD4^+^ and CD8^+^ T cell epitopes to form a single linear hybrid peptide [111,112]. Additionally, conjugating a TLR ligand with the ovalbumin (OVA) CTL epitope SIINFEKL enhances vaccine potency by increasing cellular antigen uptake, independent of binding to specific TLR receptors [113,114].

Multi-epitope vaccines can also be aligned and expressed in various vaccine constructs, such as protein, DNA, or mRNA vaccines. To accelerate the design of “string-of-beads vaccines”, a framework based on mathematical models has been developed to arrange epitopes according to their physicochemical characteristics and to determine the best linkers to connect them in sequence. This ensures the efficient expression and proteolytic processing of each epitope [115]. However, computationally designed vaccines require experimental validation for antigen expression, epitope processing and presentation, immunogenicity, and protective efficacy.

### 3.3. Mosaic Vaccines

The initial strategy for the mosaic vaccine used computational algorithms to generate artificial protein sequences that optimally cover potential T cell epitopes in globally circulating strains of HIV [116]. Studies in rhesus monkeys demonstrated that mosaic vaccines not only increased the number of distinct epitopes recognized by T cells (breadth) but also enhanced the cross-recognition of T cells with diverse variants within specific epitopes (depth) [117,118]. This approach has significant implications for developing vaccines against pathogens where antigenic variation poses a major challenge to immune protection, such as HCV [119] and influenza A (IVA) [120]. Other mosaic vaccine strategies involve targeting the same epitopes but with different compositions, thereby inducing mosaic immunity at the population level [121]. For instance, algorithms have been developed to design peptide vaccines against HCV [122] and SARS-CoV-2 [123,124] that contain multiple epitopes with high coverage across HLA populations.

### 3.4. Peptide-Pulsed DC Vaccines

Dendritic cells (DCs) are essential for mediating T cell responses by capturing, processing, and presenting antigens. They also express high levels of co-stimulatory molecules that induce immune activation [125]. DC-targeting strategies using DC vaccines have been primarily studied for cancer immunotherapy and various infectious diseases [126,127]. Typically, autologous DCs are pulsed or loaded with peptides ex vivo and then reintroduced into patients [128]. In a study involving HPV-16-positive patients, treatment with DCs pulsed with synthetic long peptides (SLPs) covering the HPV-16 oncoproteins E6 and E7 increased HPV-specific T cell responses in all participants. This approach led to 4 out of 12 patients showing no evidence of the virus in the original lesions [129,130]. Similarly, the administration of LIPO-5-DC, a DC vaccine loaded with long lipopeptides covering the Gag, Nef, and Pol epitopes of HIV, resulted in polyfunctional HIV-specific immune responses. These responses were inversely correlated with the maximum viral load observed after the cessation of highly active antiretroviral therapy (HAART) [131].

## 4. In Vitro Evaluation of Vaccine Candidates

The administration of multi-epitope vaccines can be challenging because the epitopes, when removed from the context of the whole antigen and linked together, may not follow the same processing pathways as the native pathogen. This mismatch could lead to unwanted immune responses. Mass spectrometry (MS), a useful technique for T cell epitope discovery, can be employed to measure epitope processing and presentation in vitro. This provides valuable and accurate information about the epitopes from vaccine candidates, helping determine whether individual epitopes are properly cleaved and bound to specific HLA molecules. Additionally, Jurkat-NFAT-luciferase reporter cells that express epitope-specific T cell receptors (TCRs) are ideal tools for detecting epitope processing and presentation. Luminescence is produced specifically when TCRs are activated by their cognate antigen [132]. Furthermore, a vaccine candidate should demonstrate a lack of allergic reactions and a strong ability to provoke an immune response [133]. Allergenicity can be assessed using tools such as AllerTop v2.0, AllergenFP v1.0, and AlgPred v2.0 [134,135], while antigenicity can be profiled with VaxiJen v2.0 and the ANTIGENPro server [136,137].

## 5. Animal Models for Immunogenicity and Efficiency Validation

Well-established animal models are essential for understanding disease progression, pathogenesis, and the immune response to viral infections in humans. They are also crucial for evaluating the effectiveness of vaccines and other therapeutic interventions. An optimal animal model for studying human viral infections should closely mimic the interactions between the host and the pathogen, as well as the natural progression of the disease [138]. Non-human primates (NHPs), due to their genetic similarity to humans, exhibit similar disease progression and immune responses to viral infections, making them valuable models for research [139]. However, NHPs are a limited and costly resource, which means they can only be studied in small sample sizes. In contrast, the low cost of housing mice makes it feasible for researchers to conduct large-scale studies.

### 5.1. HLA Transgenic Mice

Due to significant genetic differences between animal MHC systems and the human HLA system, the specific epitopes that can be presented vary. As a result, epitope-based vaccines designed with human T cell epitopes cannot be tested in conventional animal models such as mice, rodents, or non-human primates. Instead, HLA transgenic mice, which express human HLA molecules, are commonly used in preclinical studies to assess and optimize these vaccines for humans [140,141,142] (Table 2). This approach is effective because studies have shown a general agreement between T cell responses to peptides detected in vivo in HLA transgenic mice and in vitro responses from human peripheral blood mononuclear cells (PBMCs) [143,144]. However, discordant results can occur, especially when antigen processing is required to generate epitopes from endogenously expressed or immunized proteins. This discrepancy highlights the differences in antigen processing and presentation mechanisms between mouse and human systems [144,145].

### 5.2. Humanized Immune System Mice

Humanized immune system mice, which are engrafted with functional components of the human immune system, have been extensively studied and offer a unique opportunity to investigate human T cell responses elicited by vaccines. These models not only reveal the effects of specific vaccines but also provide insights into the development and function of human T cells following vaccination. By transplanting a population of human CD34+ cells containing hematopoietic stem cells (HSCs) into immunodeficient mice, researchers can reconstitute most major components of the human immune system, including T cells, B cells, NK cells, macrophages, and dendritic cells. This makes them valuable models for studying pathogens that infect human immune cells [146].

Such models have been widely used in virological, immunological, and pathological investigations of Epstein–Barr virus (EBV) [147,148,149,150] and HIV [151,152,153]. Various EBV vaccine candidates, such as EBV virus-like particles (VLPs) containing the EBNA1 protein [154], mRNA-based vaccines expressing T-cell-epitope-rich domains of truncated latent proteins [155], and vaccinia virus-based EBV vaccines [156], have elicited EBV-specific T cell immune responses and provided significant protection against viral infections or suppression of EBV-associated tumor progression in humanized mice. Using the NRG-hu Thy/HSC model, a vaccine regimen consisting of five HIV peptides fused to a CD40 antibody (αCD40.HIV5pep), along with poly(I:C) as an adjuvant, successfully elicited HIV-specific CD8^+^ T cell responses [157]. When administered in therapeutic settings, this vaccination significantly decreased HIV reservoirs and delayed viral rebound after the cessation of HAART [158].

However, in such humanized mouse models, the engrafted T cells are educated in the murine thymus, which lacks human HLA molecules. This results in compromised development and poor recognition of antigen peptides presented by human HLAs [159,160]. To address the inadequate interactions between human T cells and the murine environment, BLT (bone marrow, liver, thymus) mice were developed. This involves co-transplanting human fetal thymus and liver tissues along with autologous HSCs to ensure the proper education of human T cells in the human thymus and enhance their development [147,161]. BLT mice that received Gag-specific poly(lactic-co-glycolic) acid microparticles for priming, followed by a recombinant vector of replication-defective herpes simplex virus encoding the HIV Gag protein for boosting, generated broadly targeted and functionally active HIV-1-specific T cell responses [162]. Another strategy involved using poly(I:C) and STING agonist-primed dendritic cells loaded with a pool of HIV Gag peptides, which enhanced the presence of multifunctional HIV-targeted CD8^+^ T cells in lymphoid tissues while reducing the depletion of CD4^+^ T cells following HIV infection in BLT mice [163].

### 5.3. HLA Transgenic Humanized Mice

Another approach to enhancing T cell responses is the transgenic expression of human HLA genes in humanized mice, which facilitates human HLA-restricted T cell responses [164,165]. Additionally, supplementing human cytokines and growth factors can improve the engraftment and differentiation of human immune system components. For example, the expression of GM-CSF and IL-4, or treatment with FLT3-L (FMS-like tyrosine kinase 3-ligand), in humanized mice has been shown to increase the number of dendritic cells (DCs), which play a crucial role in T cell priming during vaccination [166,167]. A short carbon nanotube-based delivery formulation containing HIV glycoproteins and HIV mRNA elicited both cellular and humoral responses in humanized NSG-B2m triple mutant mice (NOD.Cg-B2^mtm1Unc^Prkdc^scid^ Il2rg^tm1Wjl/SzJ^) that express human MHC molecules (HLA-A2, HLA-DR4) and cytokines (IL-3, IL-4, IL-6, IL-7, IL-15, and GM-CSF). This led to viral clearance in 33% of the mice by 8 weeks post-infection with HIV [168].

### 5.4. Multi-System Humanized Mice

Since most human pathogens target non-hematopoietic cells, integrating these cell types into humanized immune system mouse models has significantly expanded their application in biomedical studies [169]. To model the infection and pathogenesis of hepatotropic viruses, such as HBV and HCV, humanized liver mice have been developed by engrafting human hepatocytes into mice with depleted murine liver cells. This includes various models, such as urokinase-type plasminogen activator/severe combined immunodeficiency (uPA/Scid) transgenic mice [170], FAH^−/−^RAG2^−/−^IL2RG^−/−^ (FRG) knockout mice [171,172], herpes simplex virus 1 (HSV1) thymidine kinase (TK) -NOG (Nod/Scid/IL2rg^−/−^) mice [173,174], and alpha-1-antitrypsin mutant Z protein (PiZ) transgenic NSG mice (NSG-PiZ mice) [175,176]. A liver-immune dual-humanized mouse model with the expression of HLA-A2 (A2/NSG/Fas-hu-HSC/Hep mice) has been shown to generate HBV-specific immune responses and liver injury, providing an opportunity to test vaccine strategies in a preclinical setting [177].

BLT-lung (BLT-L) mice, created by subcutaneously implanting human lung tissue into BLT mice, can efficiently control human cytomegalovirus (HCMV) and respiratory syncytial virus (RSV) infections by mounting virus-specific human antibody and T cell responses. This demonstrates the important role of human T cells in controlling HCMV and RSV infections [169,178]. A similar model, C57BL/6 Rag^−/−^γc^−/−^CD47^−/−^ (TKO)-BLT-L mice, supports SARS-CoV-2 infection and recapitulates the essential similarities and differences in how two viral variants (B.1.1.7 and 614D) infect, cause disease, and trigger immune reactions [179]. Therefore, these lung-immune dual-humanized models may serve as valuable tools for studying the immunopathology of respiratory infections and evaluating vaccine efficacy against lung pathogens.

### 5.5. TCR Repertoire Humanized Mice

It is well known that humans possess a much more diverse T cell repertoire than mice, and thymic selection plays a critical role in T cell development and TCR repertoire maturation [180,181]. To analyze the human unskewed repertoire and identify high-affinity TCRs against antigens, particularly human self-antigens such as tumors, human TCR transgenic mice (ABab mice) have been established. These mice contain complete human TCRα and TCRβ gene loci and express the entire human TCR repertoire, while not expressing murine TCRs. ABab mice were then crossed with HHDII mice, which express a chimeric HLA class I molecule composed of the α1 and α2 domains of human HLA-A*0201, the mouse H-2Db α3 transmembrane domain, and human β2m [182]. This crossbreeding produced ABabDII mice [183]. Functional antigen-specific CD8^+^ T cells were induced in ABabDII mice when they were immunized with tumor-associated antigen (TAA) peptides, and similar TCR clones were identified as those found in humans. In VelociT mice, the mouse TCRαβ variable regions were replaced along with the ectodomains of human CD4 and CD8, as well as MHC-I and MHC-II, using VelociGene technology [184]. These mice exhibited a diverse TCR repertoire and generated robust functional antigen-specific T cell responses to both acute and chronic LCMV infections. Therefore, TCR repertoire humanized mice may provide a new approach for characterizing TCR repertoire diversity specific to vaccination, allowing for a more accurate evaluation of the immunogenicity of candidate vaccines.

### 5.6. Surrogate Rat Model for HCV

Although humanized mice provide valuable insights, they are not yet capable of accurately replicating the complex situation of chronic HCV in patients, reflecting only some key characteristics [185]. To address this limitation, surrogate models of HCV infection using animal hepacivirus homologs are being explored [186]. NrHV-1, also known as rodent hepacivirus of Rattus norvegicus (RHV-rn1), causes chronic hepatotropic infection in rats, characterized by a delayed immune response and potential liver pathology, thus resembling HCV infection in humans [187]. Infection of inbred Lewis rats with RHV has shown that antiviral T cells play a vital role in determining the outcomes of hepacivirus infection in a natural host species. In this model, an adenoviral vector expressing non-structural proteins from hepacivirus triggered a protective T cell response and promoted the clearance of persistent viral infection [188]. The model was further developed using outbred Sprague–Dawley (SD) rats to better mimic human diversity and demonstrate the protective efficacy of a simian adenovirus vaccine against RHV challenge [189]. Additionally, an RHV variant expressing immune escape mutations within CD8^+^ T cell epitopes identified in rats was generated, revealing a reduced efficacy of the RHV T cell vaccine when challenged with the mutated virus [94].

## 6. Conclusions

The development of T cell epitope-based vaccines that effectively induce long-term memory T cells is a promising strategy for providing sustained protection against pathogen infections. Although technology has advanced rapidly, allowing for the discovery of pathogen epitopes and the design and optimization of immunogens, creating an effective T cell epitope-based vaccine remains a challenge. This process requires the accurate identification of epitopes restricted by various MHC molecules, a comprehensive understanding of antigen processing and presentation, and in-depth knowledge of the mechanisms involved in generating and maintaining long-lived memory T cells. Additionally, establishing appropriate animal models is essential for evaluating the vaccine’s immunogenicity, efficacy, and safety.

## Figures and Tables

**Table 1:** Advantages and disadvantages of different T cell epitope discovery approaches.

T Cell Epitope Discovery Approach	Advantages	Disadvantages
Overlapping peptide-based screening	precise identification of immunogenic peptides without requiring knowledge of the donor’s HLA profile or peptide–MHC binding characteristics	time-consuming and costly impractical for pathogens with large genomes does not account for non-canonical or cryptic epitopes
cDNA library-based screening	no constraints on epitope length identification of proteasome-generated spliced peptides	may not fully replicate the natural antigen processing machinery and MHC presentation pathways found in APCs, leading to missed or over-represented epitopes
Mass spectrometry-based screening	reduces the need for large volumes of primary samples identification of unconventional peptides	cannot identify less abundant antigens may yield false positives and false negatives, necessitating further validation through immunogenicity screening

**Table 2:** Humanization degree of different mouse models.

Model Type	TCR Repertoire	HLA	Immune System	Non-Immune System	Example Model	References
HLA transgenic	No	HLA-A2, HLA-A24	No	No	HLA-A2.1, HLA-A24.2	[140,141,142]
Humanized immune system	No	No	CD34^+^ HSC-derived immune cells	Thymus Bone marrow, liver, and thymus	NRG-hu Thy/HSC BLT	[146,147,148,149,150,151,152,153,154,155,156,157,158,159,160,161,162,163]
HLA transgenic humanized	No	HLA-A2, HLA-DR4	CD34^+^ HSC-derived immune cells	No	NOD.Cg-B2^mtm1Unc^Prkdc^scid^ Il2rg^tm1Wjl/SzJ^	[164,165,166,167,168]
Multi-system humanized	No	HLA-A2	CD34^+^ HSC-derived immune cells	Liver Lung	A2/NSG/Fas-hu-HSC/Hep BLT-L, TKO-BLT-L	[169,170,171,172,173,174,175,176,177,178,179]
TCR repertoire humanized	Human TCRα and TCRβ gene loci	HLA-A2, HLA-DR4, HLA-DR2	No	No	ABabDII ABabDR4 VelociT	[180,181,182,183,184]
